# RETRACTED: Léon et al. 6-Pyrazolylpurine as an Artificial Nucleobase for Metal-Mediated Base Pairing in DNA Duplexes. *Int. J. Mol. Sci.* 2016, *17*, 554

**DOI:** 10.3390/ijms26115409

**Published:** 2025-06-05

**Authors:** J. Christian Léon, Indranil Sinha, Jens Müller

**Affiliations:** 1 Institut für Anorganische und Analytische Chemie, Westfälische Wilhelms-Universität Münster, Corrensstraße 30, 48149 Münster, Germany; leonchristian458@gmail.com (J.C.L.); indranilsinha14@gmail.com (I.S.); 2 NRW Graduate School of Chemistry, Westfälische Wilhelms-Universität Münster, Corrensstraße 30, 48149 Münster, Germany

The Journal retracts the article “6-Pyrazolylpurine as an artificial nucleobase for metal-mediated base pairing in DNA duplexes” [1] cited above.

Following publication, the authors contacted the Editorial Office to raise concerns relating to identity of the oligonucleotides used in that publication [1], as a result of an inadvertent error. In particular, subsequent attempts to reproduce their synthesis indicated that the artificial nucleobase 6-pyrazolylpurine had decomposed upon or after incorporation into the oligonucleotides, i.e., prior to its study in the DNA duplexes. In the meantime, correct results and several reference measurements have been reported elsewhere [2].

Adhering to our standard procedure, an investigation was conducted by the Editorial Office and Editorial Board that confirmed weaknesses in the experimental evidence provided in the article. Given the impact of this issue on the validity of results, the Editorial Board and the authors have determined that the overall findings could not be relied upon and decided to retract this publication [1], as per MDPI’s retraction policy (https://www.mdpi.com/ethics#_bookmark30).

This retraction was approved by the Editor-in-Chief of the journal *IJMS.*

The authors agreed to this retraction.
